# Absence of associations with prefrontal cortex and cerebellum may link to early language and social deficits in preschool children with ASD

**DOI:** 10.3389/fpsyt.2023.1144993

**Published:** 2023-05-04

**Authors:** Jing Xin, Kaiyu Huang, Aiwen Yi, Ziyu Feng, Heng Liu, Xiaoqing Liu, Lili Liang, Qingshan Huang, Yaqiong Xiao

**Affiliations:** ^1^Foshan Clinical Medical School, Guangzhou University of Chinese Medicine, Foshan, China; ^2^Center for Language and Brain, Shenzhen Institute of Neuroscience, Shenzhen, China; ^3^Department of Radiology, Affiliated Hospital of Zunyi Medical University, Medical Imaging Center of Guizhou Province, Zunyi, China

**Keywords:** autism spectrum disorder, preschool children, brain-behavior correlation, language and social abilities, prefrontal cortex, cerebellum

## Abstract

**Introduction:**

Autism spectrum disorder (ASD) is a complex developmental disorder, characterized by language and social deficits that begin to appear in the first years of life. Research in preschool children with ASD has consistently reported increased global brain volume and abnormal cortical patterns, and the brain structure abnormalities have also been found to be clinically and behaviorally relevant. However, little is known regarding the associations between brain structure abnormalities and early language and social deficits in preschool children with ASD.

**Methods:**

In this study, we collected magnetic resonance imaging (MRI) data from a cohort of Chinese preschool children with and without ASD (24 ASD/20 non-ASD) aged 12–52 months, explored group differences in brain gray matter (GM) volume, and examined associations between regional GM volume and early language and social abilities in these two groups, separately.

**Results:**

We observed significantly greater global GM volume in children with ASD as compared to those without ASD, but there were no regional GM volume differences between these two groups. For children without ASD, GM volume in bilateral prefrontal cortex and cerebellum was significantly correlated with language scores; GM volume in bilateral prefrontal cortex was significantly correlated with social scores. No significant correlations were found in children with ASD.

**Discussion:**

Our data demonstrate correlations of regional GM volume with early language and social abilities in preschool children without ASD, and the absence of these associations appear to underlie language and social deficits in children with ASD. These findings provide novel evidence for the neuroanatomical basis associated with language and social abilities in preschool children with and without ASD, which promotes a better understanding of early deficits in language and social functions in ASD.

## Introduction

Autism spectrum disorder (ASD) has been widely recognized as a complex neurodevelopmental disorder, characterized by social affective and communication deficits that begin to appear in the early years of life. While the neural mechanism of ASD is still elusive, consistent and robust findings from magnetic resonance imaging (MRI) studies suggest an atypical and dynamic neurodevelopmental trajectory of brain structure in ASD individuals, i.e., early brain overgrowth and slow brain development in late childhood (1–4). In fact, the theory of early brain overgrowth in young children with ASD aged 2–6 years has been supported by findings from multiple independent samples in both cross-sectional (5–9) and longitudinal studies (10). Further, a number of studies have shown that a wide range of brain regions are involved in abnormal early brain overgrowth in children with ASD. A meta-analysis reported that children with ASD had significant gray matter (GM) volume increases in the right angular gyrus, right inferior temporal gyrus (ITG), left superior and middle frontal gyrus, left precuneus, and left inferior occipital gyrus (11). Another meta-analysis reported increased GM volume in bilateral fusiform gyrus, right cingulate, and insula in children and adolescents with ASD (12). A recent study with a group of children with ASD aged 3–7 years found greater GM volume in left superior temporal gyrus and the left postcentral gyrus (8). However, a few studies found no differences in total or regional brain volume in young children with ASD as compared to controls (13–16). These discrepancies may be due to different samples sizes, age ranges, and populations, and thus further studies are still needed to elucidate the underlying neuroanatomical basis in young children with ASD.

It has been suggested that ASD is a prenatal multistage, multi-process disorder that begins in the first trimester and continues throughout second and third trimesters and postnatal stages (17, 18), which thus leads to atypical trajectories of language and social abilities from the very beginning. Indeed, as compared to typically developing (TD) toddlers and ASD children with better language outcome, ASD children with poor language outcome had atypical cortical patterns, which can be identified in the first few years of life (19). Neural correlates of early language and social deficits have also been found to be associated with abnormal inter-hemispheric functional connectivity between language-related regions (20), reduced neural activation in language regions (21–23), and abnormal connectivity pattern between the default mode network and visual network (24, 25).

While evidence is still limited, a few studies have examined the behavioral relevance of atypical brain structure in young children with ASD. For example, research by Retico et al. (16) identified increased GM volume in bilateral superior frontal gyrus, bilateral superior temporal gyrus, and bilateral precuneus, but these GM volume changes did not associate with the severity of ASD symptoms. Wang et al. (8) investigated the relationship between brain structural alterations and core symptoms of young children with ASD, but did not find any significant correlations between GM volume of altered brain regions and severity of ASD symptoms. When using the altered brain regions as seed regions for the functional connectivity analysis, the degree of connectivity alterations was related to the symptom severity in children with ASD (8). A study by Cai et al. (26) reported greater GM volume in left ITG in children with both low and high functioning ASD, and demonstrated a negative correlation between left ITG and repetitive behavior in children with high functioning ASD. When dividing ASD children aged 3–6 years into verbal and non-verbal subgroups, significantly reduced total cerebellar white matter (WM) volume was found in non-verbal ASD patients, suggesting there were associations between brain structure abnormalities and clinical features of ASD (6). These findings suggest potential neural basis underlying atypical cognitive development in young children with ASD. So far, however, no evidence has shown whether and how brain structure abnormalities are related to early language and social deficits in young children with ASD.

To fill in this research gap, in the present study, we first explored cortical GM volume differences in a cohort of Chinese preschool children aged 12–52 months with and without ASD. We expected there would be greater global and regional GM volume in children with ASD as compared to those without ASD. Next, we examined associations between regional GM volume and early language and social abilities in the ASD and non-ASD groups, separately. We expected to observe significant but different correlation patterns for language and social abilities in the non-ASD group, and for children with ASD, we expected there might be different correlation patterns as a reflection of early language and social deficits in this group.

## Materials and methods

### Participants

In this study, we recruited 35 outpatients with and without ASD (24 ASD/11 non-ASD) aged 12–52 months from the Foshan Fosun Chancheng Hospital, Foshan, China between October 2021 and May 2022, and 9 children without ASD aged 19–38 months from the Affiliated Hospital of Zunyi Medical University, Zunyi, China between March 2022 and July 2022. A total of 35 participants completed the Chinese version of Gesell Development Diagnosis Scale (GDDS), which measures children’s development in different domains including personal-social behavior, language, fine motor, gross motor, and adaptive behavior (27). All of the ASD patients (18 M/6 F; mean age: 36.8 ± 9.2 months, range: 24–51 months) fulfilled DSM-V criteria for ASD as diagnosed by clinical interview and completed the Autism Diagnostic Observation Schedule (ADOS model 1, 2) (*n* = 9), Childhood Autism Rating Scale (CARS) (*n* = 21), or Autism Behavior Checklist (ABC) (*n* = 18). Non-ASD children (17 M/3 F; mean age: 29.4 ± 11.5 months, range: 12–52 months) were either TD or had developmental delays but without a diagnosis of ASD based on their GDDS, CARS, or ABC scores, or other clinical measures (scores were not available for research). ADOS, CARS, and GDDS were administrated by trained clinicians, and ABC was completed by parents or guardians of the participants. For detailed demographic and clinical information, see Table 1. All children were native Mandarin or Cantonese speakers with normal hearing and no family history of mental or psychiatrical disorders. This study was approved by the Medical Ethics Committee of Foshan Fosun Chancheng Hospital and the Affiliated Hospital of Zunyi Medical University, separately. Informed consent was obtained from parents or guardians of participants.

**TABLE 1:** Demographic details and clinical and behavioral testing scores.

	ASD (*n* = 24)		Non-ASD (*n* = 20)		ASD vs. non-ASD	
	Mean ± SD	Range	Mean ± SD	Range	*t*-Value	*p*-Value
Age (months)	36.8 ± 9.2	24–51	29.4 ± 11.5	12–52	2.29	0.03
Gender (M/F)	18/6		17/3		0.2^a^	0.66
**Gesell subscale scores^b^**						
Gross motor	71 ± 10.79	47.8–91	87.92 ± 11.8	70–105.5	−4.30	<0.001
Fine motor	69.5 ± 13.3	48.3–94.6	84.39 ± 8.5	75.3–97	−4.04	<0.001
Personal-social behavior	51.68 ± 10.52	31–66.7	73.87 ± 8.52	59.1–89.8	−6.87	<0.001
Language	42.26 ± 8.9	26.6–60.9	61.07 ± 10.29	47–90.8	−5.59	<0.001
Adaptive behavior	61.41 ± 14.44	35.6–93.7	78.45 ± 13.19	60.4–100	−3.60	0.001
Gesell total	59.4 ± 9.48	39.3–74.4	77.14 ± 7.89	66.6–92.7	−6.01	<0.001
ADOS^c^ social and communication	14.11 ± 3.14	9–19				
ADOS RRB	2.11 ± 1.36	1–4				
CARS total^d^	33.95 ± 4.33	30–42	22.67 ± 5.13	17–27	3.63	0.051
ABC total^e^	62.44 ± 22.65	31–111	22 ± 16.07	6–55	5.61	<0.001

ASD, autism spectrum disorder; RRB, restricted and repetitive behavior; ADOS, autism diagnostic observation schedule; CARS, childhood autism rating scale; ABC, autism behavior checklist.

^a^The value was from the Chi-squared test.

^b^Gesell was successfully administrated in 21 ASD children (15 M/6 F) and 14 non-ASD children (12 M/2 F).

^c^ADOS was successfully administrated in 9 children with ASD (8 M/1 F).

^d^CARS was successfully administrated in 21 children with ASD (15 M/6 F) and 3 children without ASD (2 M/1 F).

^e^ABC was completed by parents or guardians of 18 children with ASD (12 M/6 F) and 11 children without ASD (9 M/2 F).

### MRI data acquisition

Before MRI scanning, all of the participants were administered 0.5% chloral hydrate 0.5 ml/kg (maximum dose 10 ml) orally to induce and maintain sleep. All participants continued sleeping during scanning and were not stimulated.

Most of structural MRI data (*n* = 35) were collected on a 3.0T SEMENS Skyra scanner at the Foshan Chancheng Hospital, Foshan, China using a T1-weighted MPRAGE sequence (TE = 2.98 ms, TR = 2,300 ms, resolution = 1.0 mm × 1.0 mm × 1.0 mm, space gap = 0 mm, flip angle = 9°, 144 slices). The remaining MRI data (*n* = 9) were collected on a 3.0T GE 750 scanner using a T1-weighted MPRAGE sequence (TE = 3.2 ms, TR = 8.4 ms, FOV = 256 mm, Matrix = 256 × 256 mm, resolution = 1.0 mm × 1.0 mm × 1.0 mm, space gap = 0 mm, flip angle = 15°, 146 slices). The scanner information was coded as 1 and 2 and added as a categorical variable in the statistical analyses.

### MRI data processing

Prior to processing, all MRI images were visually inspected and then reoriented to the standard anterior commissure-posterior commissure plane. MRI data were processed with the voxel-based morphometry (VBM) pipeline using the Computational Anatomy Toolbox (CAT12^1^), an extension of Statistical Parametric Mapping (SPM12^2^), running in Matlab R2020a (MathWorks, Natick, MA, USA). To minimize the potential confounds introduced by different brain sizes and tissues between young children and adults (28), custom pediatric tissue probability maps and DARTEL templates were created with the CerebroMatic (COM) toolbox^3^ (29, 30). The COM toolbox provides regression parameters modeled with 1,914 healthy participants aged 13 months to 75 years. Specifically, the COM toolbox can be used to generate the custom tissue probability maps by matching sample demographics to parameters that influence brain structure using a flexible non-parametric approach: multivariate adaptive regression splines (29). The custom DARTEL templates can also be created using the COM toolbox, which matches sample demographics to a second set of regression parameters derived from 1,919 participants in the same databases (30). Here, age and sex of each participant and field strength of the scanners were entered into the COM toolbox to create the custom tissue probability maps and DARTEL templates, separately.

For the VBM analysis, MRI images were segmented into GM, WM, and cerebrospinal fluid (CSF) maps. The segmented data were affine registered to the custom pediatric tissue probability maps. Extracted GM maps were spatially normalized to the study-specific pediatric template using the DARTEL registration. Then, GM images were modulated with Jacobian determinants from the normalization procedure to preserve regional volumes. The modulated normalized non-linear images were checked for sample homogeneity and no outlier images were detected. The GM images were smoothed with an 8 mm full-width at half-maximum smoothing kernel. The voxel size of processed images was 1.5 mm × 1.5 mm × 1.5 mm. The total GM, WM, CSF, and intracranial volume (TIV) were extracted for each participant in the CAT12.

### Statistical analysis

All subjects (24 ASD/20 non-ASD) were included in the group analyses to identify group differences in total brain volume and regional GM volume, and 35 subjects (21 ASD/14 non-ASD) who had usable Gesell language and personal-social behavior scores were included in the brain-behavior correlation analysis.

#### Group differences in total brain volume

Group differences were evaluated for the total volume of GM, WM, CSF, and TIV. Specifically, two-tailed independent *t*-tests were applied to identify any significant difference between children with and without ASD in global brain volume.

#### Group differences in regional GM volume

We conducted the whole brain voxel-wise analysis to examine the group differences in GM maps using the ‘y_TTest2_Image’ function in DPABI (a toolbox for Data Processing and Analysis for Brain Imaging^4^) (31), with age, quadratic age, gender, TIV, and scanner information as covariates of no interest. A group-specific binary GM mask was used for the group analysis, which was created with image calculator function in SPM12. Results were corrected for multiple comparisons at the cluster-level using the Gaussian random field (GRF) theory, a family-wise error (FWE) correction approach, in which *t* maps were converted to *z* maps and significant clusters met the following thresholds: voxel-wise *p* = 0.005, cluster size >1,300 voxels (cluster-wise *p* < 0.05, two-tailed; *Z* > 2.8).

#### Brain-behavior correlation analysis

Further, we examined the relationships between cortical GM volume and children’s language and social abilities as measured by Gesell language and personal-social behavior subtests in the ASD and non-ASD groups, separately. Specifically, using the “y_Correlation_Image” function in DPABI, we conducted the correlation analysis between whole-brain GM maps and Gesell language and personal-social behavior scores for ASD and non-ASD groups separately, controlling for age, quadratic age, gender, and scanner information (only for the non-ASD group). The resulting correlation maps were converted to the *z* maps and corrected for multiple comparisons at the cluster-level using the GRF correction (voxel-wise *p* < 0.005, cluster size >1,300 voxels, cluster-wise *p* < 0.05, *Z* > 2.8, two-tailed). Finally, all the significant clusters were visualized with the BrainNet Viewer.^5^

## Results

### Group differences in total volume of GM, WM, CSF, and TIV between ASD and non-ASD groups

**FIGURE 1:**
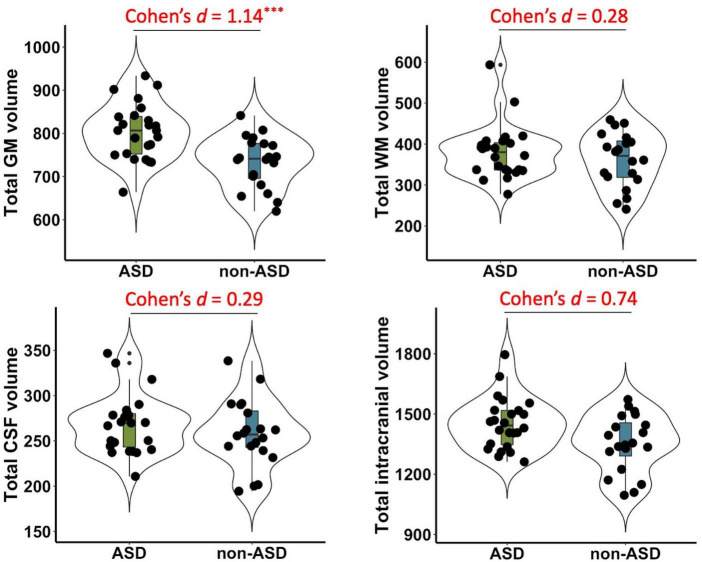
Group differences in total gray matter (GM), white matter (WM), cerebrospinal fluid (CSF), and intracranial volume between ASD and non-ASD groups. Significant differences were observed in the GM volume (*p* < 0.001), but not in total WM (*p* = 0.36), CSF (*p* = 0.35), or total intracranial volume (*p* = 0.02) after FDR correction. Cohen’s *d*, standardized effect sizes for group comparisons. ^***^p < 0.001.

Significant group differences between ASD and non-ASD groups were observed in the total GM volume (*t*(42) = 3.79, *p* < 0.001, Cohen’s *d* = 1.14, 95% CI = [0.48, 1.8]), but not in total WM (*t*(42) = 0.93, *p* = 0.36, Cohen’s *d* = 0.28, 95% CI = [−0.33, 0.9]), CSF volume (*t*(42) = 0.94, *p* = 0.35, Cohen’s *d* = 0.29, 95% CI = [−0.32, 0.9]), or TIV (*t*(42) = 2.41, *p* = 0.02, Cohen’s *d* = 0.74, 95% CI = [0.11, 1.37]) after FDR correction (see Figure 1).

### No significant differences in regional GM volume between ASD and non-ASD groups

The whole-brain voxel-wise analysis did not show any significant differences in regional GM volume between ASD and non-ASD groups after correcting for multiple comparisons with the GRF method (voxel-wise *p* = 0.005, cluster size >1,300 voxels, cluster-wise *p* < 0.05, *Z* > 2.8, two tailed).

### Correlations between regional GM volume and language and social abilities in non-ASD children

**FIGURE 2:**
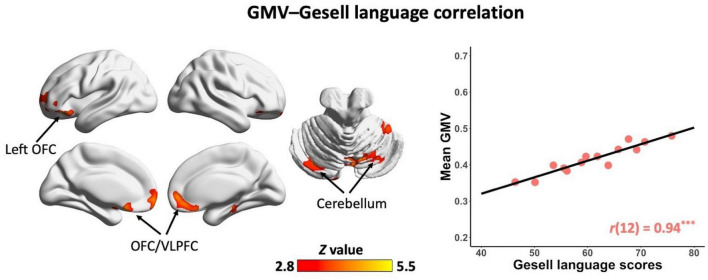
Clusters showing significant correlations between gray matter volume and Gesell language scores in the non-ASD group (GRF corrected; voxel-wise *p* = 0.005, cluster size >1,300 voxels, cluster-wise *p* < 0.05, *Z* > 2.8, two-tailed). The scatterplot and *r* value present significant correlations between mean gray matter volume of significant clusters (i.e., bilateral OFC/VLPFC and bilateral cerebellum) and Gesell language scores, controlling for age, quadratic age, gender, and scanner information. OFC, orbitofrontal cortex; VLPFC, ventrolateral prefrontal cortex; GMV, gray matter volume. ^***^p < 0.001.

**FIGURE 3:**
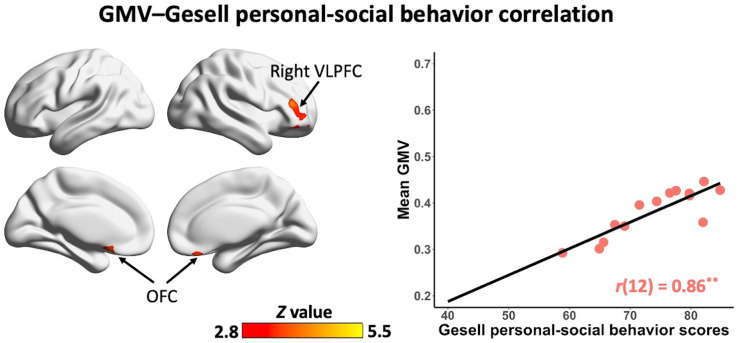
Clusters showing significant correlations between gray matter volume and Gesell personal-social behavior scores in the non-ASD group (GRF corrected; voxel-wise *p* = 0.005, cluster size >1,300 voxels, cluster-wise *p* < 0.05, *Z* > 2.8, two-tailed). The scatterplot and *r* value present significant correlations between mean gray matter volume of significant clusters (i.e., bilateral OFC, right VLPFC) and Gesell personal-social behavior scores, controlling for age, quadratic age, gender, and scanner information. OFC, orbitofrontal cortex; VLPFC, ventrolateral prefrontal cortex; GMV, gray matter volume. ^**^p < 0.01.

In the non-ASD group, GM volume in bilateral prefrontal cortex [including bilateral orbitofrontal cortex (OFC) and right ventrolateral prefrontal cortex (VLPFC)] [*r*(12) = 0.87, *p* = 0.001] and cerebellum [left cerebellum: *r*(12) = 0.97, *p* < 0.001; right cerebellum: *r*(12) = 0.85, *p* = 0.002] was significantly correlated with Gesell language scores (Figure 2 and Table 2). GM volume in bilateral prefrontal cortex [right VLPFC: *r*(12) = 0.85, *p* = 0.002; bilateral OFC: *r*(12) = 0.83, *p* = 0.003] was significantly correlated with Gesell personal-social behavior scores (Figure 3 and Table 2). Consistent with the brain-behavior correlation analysis, *p* values presented here were calculated using the partial correlation analysis, controlling for age, quadratic age, gender, and scanner information. All the resulting clusters were corrected for multiple comparisons (voxel-wise *p* = 0.005, cluster size >1,300 voxels, cluster-wise *p* < 0.05, *Z* > 2.8, GRF corrected). However, there were no significant associations between GM volume and Gesell language or personal-social behavior scores in the ASD group.

**TABLE 2:** Clusters showing significant correlations with Gesell language and personal-social behavior scores in the non-ASD group.

			Peak MNI				
Gesell measure	Region	Brodmann area	*X*	*Y*	*Z*	Peak intensity (*Z* value)	Number of voxels
Language	Bilateral OFC, right VLPFC	BA 11/10/47	−2	18	−24	5.22	9,431
	Right cerebellum		15	−69	−24	4.93	6,578
	Left cerebellum		−12	−48	−56	4.1	1,443
Personal-social behavior	Right VLPFC	BA 11/46	36	38	12	4.6	1,894
	Bilateral OFC	BA 11	−3	18	−32	4.2	1,673

OFC, orbitofrontal cortex; VLPFC, ventrolateral prefrontal cortex; BA, Brodmann area.

## Discussion

In the present study, we examined the brain structure differences between preschool children with and without ASD and the associations between brain structure and early language and social abilities in these two groups, separately. As expected, there was significant group difference in total GM volume, but we did not observe any regional GM volume differences between children with and without ASD. In children without ASD, we found significant associations between GM volume in bilateral prefrontal cortex (i.e., OFC and VLPFC) and cerebellum and Gesell language scores, and between GM volume in bilateral prefrontal cortex (i.e., OFC and VLPFC) and Gesell personal-social behavior scores. However, no brain-behavior correlations were found in children with ASD. These findings demonstrate significant correlations of regional GM volume with early language and social abilities in preschool children without ASD, and the absence of these associations in children with ASD appears to reflect their atypical language and social development.

One of the key findings here is the significant association of GM volume in bilateral prefrontal cortex (i.e., OFC and VLPFC) and cerebellum with language ability in children without ASD. The cerebellum has been widely recognized as a region related to a variety of cognitive functions such as language (32–36). The association between GM volume in bilateral cerebellum and language ability further supports the involvement of cerebellum in language function. Previous research has also shown that cerebellar abnormalities underlie language deficits in ASD (37–39). Moreover, atypical activation, connectivity, and structure in the circuits between cerebellum and language-related cortical regions are associated with language impairments in ASD (39, 40). Here, we observed associations of cerebellum and prefrontal cortex with language ability in children without ASD, implying the important role of the circuits between cerebellum and prefrontal regions in language development. On the contrary, this association was absent in children with ASD, suggesting abnormal structure in cerebellum and disruptions of the circuits between cerebellum and prefrontal regions may underlie early language impairments in ASD.

Interestingly, there were also significant correlations of GM volume in bilateral prefrontal cortex (i.e., OFC and VLPFC) and social ability in children without ASD. Both OFC and VLPFC are well-established regions that are critical for social cognition (41–43). A number of studies have shown that the disruptions of these regions are linked to social deficits in ASD (44–46). A recent study suggests that abnormal connectivity of OFC with neighboring regions may provide the anatomic basis for the disrupted signal transmission for social and emotion functions in ASD (47). Here, the association of bilateral OFC with social ability in children without ASD further suggests the important role that the OFC plays in typical social development. Notably, the correlations of regional GM volume and social ability in the non-ASD group did not involve the cerebellum. Previous studies have shown the cerebellum is related to social cognition [e.g., (33, 48, 49)] and the circuits between the cerebellum and social-related cortical regions are associated with social impairments in ASD (39, 40). It might be due to the small sample size in the present study, and future studies are needed to investigate the involvement of the cerebellum in typical social development in young children.

Our data demonstrated significantly increased total GM volume in children with ASD as compared to those without ASD, which is consistent with previous studies reporting enlarged brain volume in young children with ASD vs. matched controls (5, 9, 50, 51). However, we did not observe significant differences in total WM or CSF volume. In fact, there were also a few studies that did not find differences in brain volume between ASD and non-ASD groups (13–16), which suggests the findings are inconsistent regarding the neuroanatomical basis related to ASD. While it has been extensively reported there are altered cortical patterns in children with ASD, especially in frontal and temporal regions (8, 11, 16, 52–55), we did not find any significant differences in regional GM volume between children with and without ASD. These findings imply the neuroanatomical heterogeneity of ASD, which may underlie different trajectories of severity of symptoms and cognitive functions in individuals with ASD (56, 57). Thus, even though enlarged GM volume and abnormal anatomical organizations have been consistently reported as a core neural feature in early years of ASD, conclusions still cannot be made. Future studies with larger sample sizes are needed to identify potential subtypes of ASD with different brain anatomical features, which may facilitate prognosis and individual treatment for children with ASD.

A few limitations are worth noting when interpreting the results reported in this study. First, we included children without ASD instead of TD children as the control group. These non-ASD children had varying language and social abilities, which may contribute to the significant brain-behavior correlations. And the associations in children without ASD may not necessarily be generalized to TD children. Future research should include TD children to confirm these findings observed in non-ASD children. Second, sample sizes in both ASD and non-ASD groups are relatively small given the difficulties of MRI data collection in children aged 2–6 years, especially the samples with available behavioral measures. These findings need to be replicated in studies with large sample sizes. Third, the samples included in this study were collected in two sites using two different MRI scanners. Though we included the scanner information (coded as 1 and 2) as a covariate of no interest in the statistical analyses, potential effects caused by different scanners and scan protocols may not be ruled out. Thus, MRI data collected in the same scanner with the same scan protocols should be considered in the future studies. Fourth, the ASD group had lower cognitive functions as compared to the non-ASD group. It is possible that the greater global GM volume in the ASD group is due to lower cognitive ability instead of ASD *per se*. Future research should control for the cognitive ability (e.g., IQ or Developmental Quotient) when examining ASD-related cortical morphometry differences.

## Conclusion

In sum, we observed significant enhanced total GM volume in a cohort of Chinese preschool children with ASD as compared to those without ASD. However, opposite to our hypothesis, there were no significant differences in regional GM volume between ASD and non-ASD groups. In children without ASD, we observed significant associations of GM volume in bilateral prefrontal cortex (i.e., OFC and VLPFC) and cerebellum with Gesell language scores, and associations of GM volume in bilateral prefrontal cortex (i.e., OFC and VLPFC) with Gesell personal-social behavior scores, but no significant correlations were found in children with ASD. These results suggest correlations of regional GM volume with early language and social abilities in preschool children without ASD, and the absence of these associations appear to underlie early language and social deficits in ASD. These findings provide novel insights into the neural basis of language and social abilities in preschool children with and without ASD.

## Data availability statement

The datasets presented in this study can be found in online repositories. The names of the repository/repositories and accession number(s) can be found below: https://github.com/Yaqiongxiao/asdGM.language.social.

## Ethics statement

The studies involving human participants were reviewed and approved by the Medical Ethics Committee of Foshan Fosun Chancheng Hospital and the Affiliated Hospital of Zunyi Medical University. Written informed consent to participate in this study was provided by the participants’ legal guardian/next of kin.

## Author contributions

YX and JX contributed to the conception and design of this study. JX, AY, LL, HL, XL, and QH contributed to the clinical and MRI data acquisition. YX and KH contributed to the data analysis. YX drafted the manuscript. All authors contributed to the manuscript edits and revisions.

## Footnotes

1
https://neuro-jena.github.io/cat/

2
https://www.fil.ion.ucl.ac.uk/spm/software/spm12/

3
https://www.medizin.uni-tuebingen.de/kinder/en/research/neuroimaging/software/

4
https://rfmri.org/dpabi

5
http://www.nitrc.org/projects/bnv/
